# Supplementary material for: Real-time 3D analysis during electron tomography using tomviz
Source: Nat Commun. 2022 Aug 1;13:4458. doi: 10.1038/s41467-022-32046-0 (PMC9343612; doi:10.1038/s41467-022-32046-0)
Supplement: Supplementary file 1 — Supplementary Information [file 41467_2022_32046_MOESM1_ESM.pdf]

Supplementary Information for

Real-Time 3D Analysis During Electron Tomography using tomviz

Jonathan Schwartz, Chris Harris, Jacob Pietryga, Huihuo Zheng, Prashant Kumar, Anastasiia Visheratina, Nicholas Kotov, Brianna Major, Patrick Avery, Peter Ercius, Utkarsh Ayachit, Berk Geveci, David Muller, Alessandro Genova, Yi Jiang, Marcus Hanwell, and Robert Hovden

S1 Real-Time Cryo Electron Tomography Demonstration on a Cd-Cys Nanoparticle

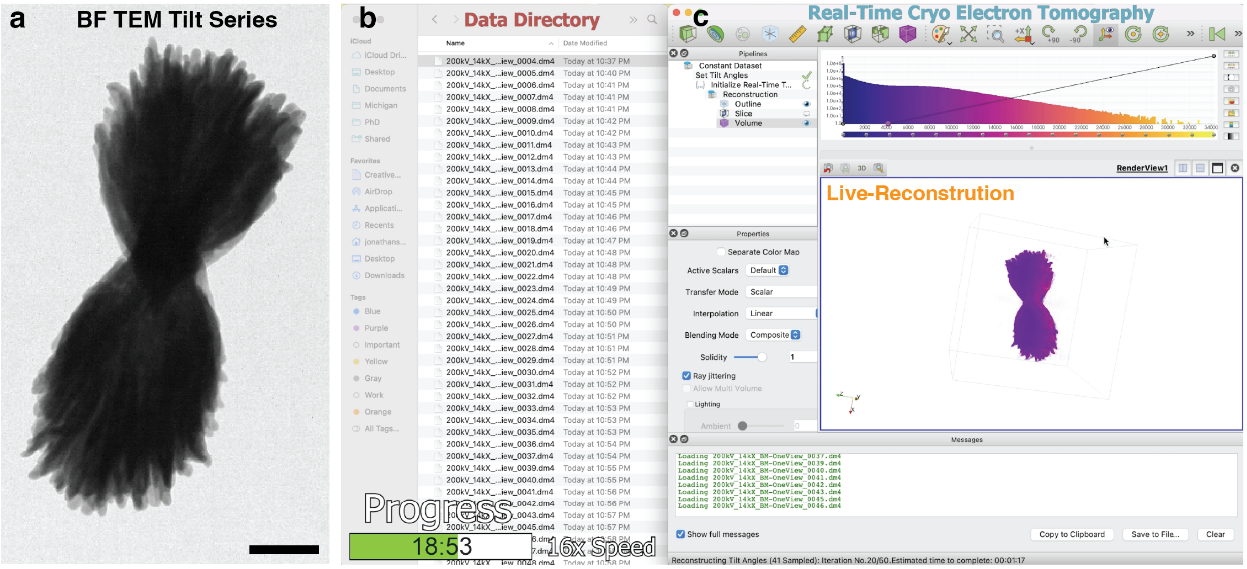

Fig. S1 | a) Bright field projection images collected during the real-time cryo electron tomography experiment. Scale bar, 500 nm. b-c) A screenshot from Supplementary Video 6 demonstrating an experimental real-time cryo-electron tomography reconstruction. The data directory indicates when a new projection images was collected from the microscope, which is intermittently read by tomviz for processing.

S2 Real-Time Simulation for the Raw FePt Nanoparticle

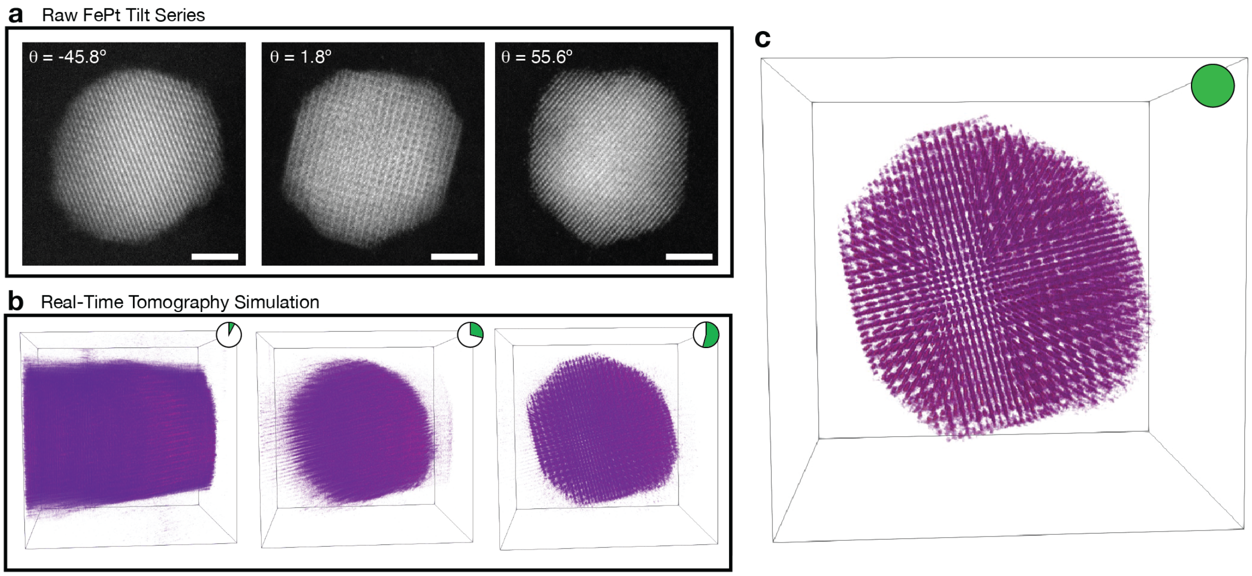

Fig. S2 | a) The raw FePt tomographic projection images used for the real-time tomography simulation. Scale bar, 2 nm. b) As the simulation progresses, the reconstruction quality improves and atoms become visible as early as 50% of the experiment. c) An atomic-resolution tomogram is available after all the projections are reconstructed.

S3 Comparison of center of mass alignment and cross correlation for non-isolated objects

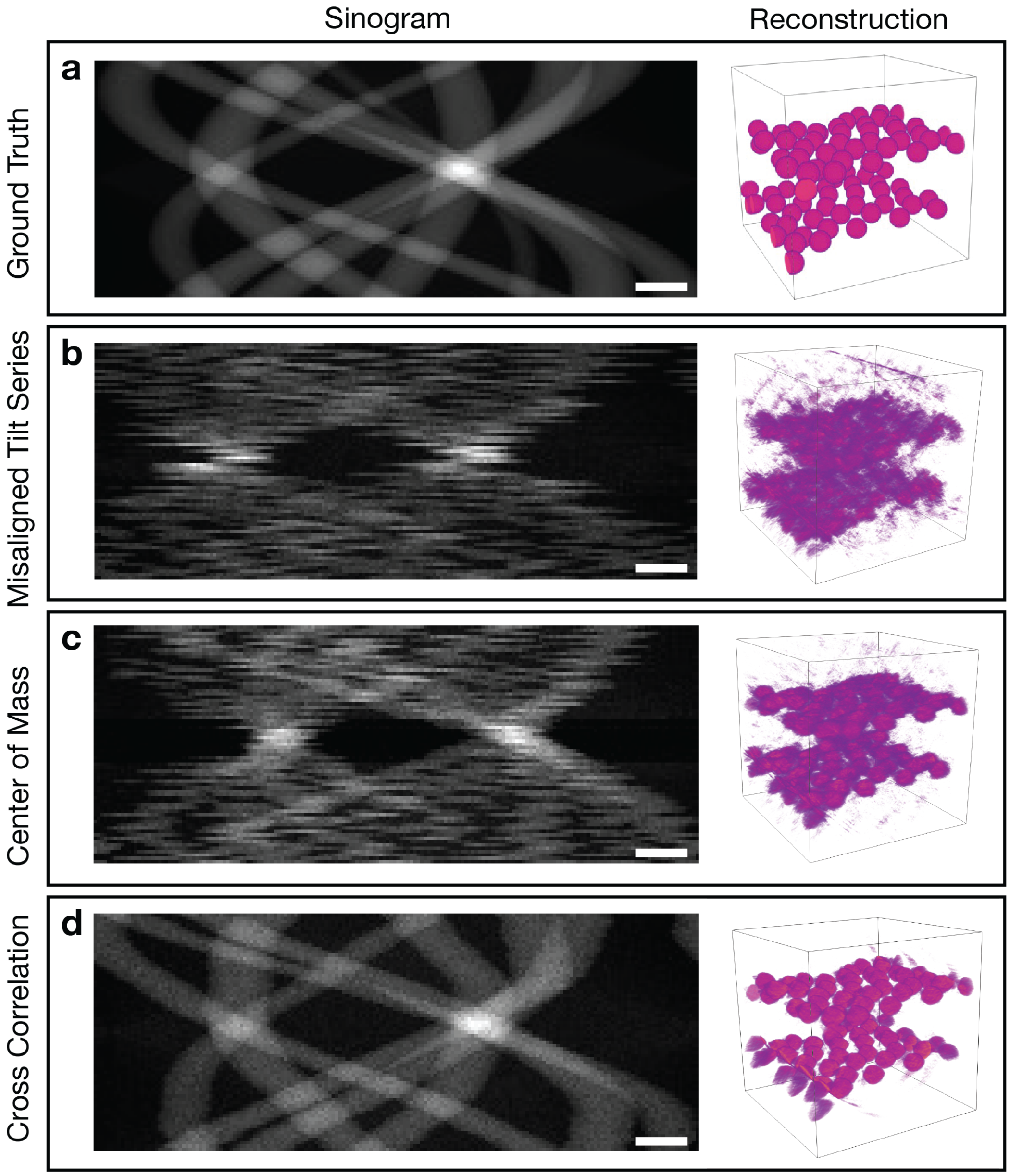

Fig. S3 | a) The phantom test object consisting two layers of nanoparticles inspired by an experimental Fe_3O_4 material system. b) The sinogram that corrupted by Poisson noise and misaligned by random translational image shifts. c-d) The sinograms aligned by center of mass and cross correlation and its resulting reconstructions. Scale bar, 10 nm.
