# Supplementary material for: Real-time 3D analysis during electron tomography using tomviz
Source: Nat Commun. 2022 Aug 1;13:4458. doi: 10.1038/s41467-022-32046-0 (PMC9343612; doi:10.1038/s41467-022-32046-0)
Supplement: Supplementary file 3 — Description of Additional Supplementary Files [file 41467_2022_32046_MOESM3_ESM.pdf]

Description of Additional Supplementary Files

Jonathan Schwartz, Chris Harris, Jacob Pietryga, Huihuo Zheng, Prashant Kumar, Anastasiia Visheratina, Nicholas Kotov, Brianna Major, Patrick Avery, Peter Ercius, Utkarsh Ayachit, Berk Geveci, David Muller, Alessandro Genova, Yi Jiang, Marcus Hanwell, and Robert Hovden

Supplementary Dataset 1: Projection Images from the Real-Time Cd-Cyst Experiment

The tilt series of a twisted Cd-Cyst nanoparticle from a real-time tomography experiment. Tomographic data was acquired using a Talos F200X (Thermo Fisher) operated at 200kV with a 10.5 mrad semi-convergence angle using an annular dark field detector. A tilt series of 72 projections was acquired over a ± 70° range at 2° intervals using a high angle annular dark-field detector. The scale in each image is 11.97 nm per pixel with total image dimension of 300 by 300 pixels (32 bit).

Supplementary Video 1: Live 3D Visualization During Tomographic Experiments with Electron Microscopes

An experimental demonstration of the real-time tomography experiment that runs simultaneously as users collect data off the microscope.

Supplementary Video 2: Live 3D Visualization During Tomographic Experiments (Simulated Demonstration)

A simulated demonstration of a real-time highlighting the process that runs on tomviz.

Supplementary Video 3: Live 3D Visualization During Reconstruction

A tutorial for enabling live reconstruction updates on tomviz.

Supplementary Video 4: Real-Time 3D Visualization of SIRT Reconstruction of Cobalt Phosphate Hyperbranched Nanoparticle

A real-time reconstruction of cobalt phosphate hyberbranched nanoparticles produced with the simultaneous iterative reconstruction technique algorithm.

Supplementary Video 5: Real-Time 3D Visualization of TVmin Reconstruction of Iron Nanoparticle

A real-time reconstruction of iron plantinum nanoparticles produced with the total variation minimization algorithm.

Supplementary Video 6: Real-Time 3D Visualization of Weighted Back Projection Reconstruction of Platinum Nanoparticles on Carbon Nanowire

A real-time reconstruction of plantinum nanoparticles on a carbon nanowire produced with the weighted back projection algorithm.

Supplementary Video 7: Real-Time Cryo Electron Tomography Demonstration on a Cd-Cyst Nanoparticle

An experimental demonstration of a cryo-BF TEM real-time tomography experiment on the Cd-Cyst nanoparticle.
